# Network Simulations Reveal Molecular Signatures of Vulnerability to Age-Dependent Stress and Tau Accumulation

**DOI:** 10.3389/fmolb.2020.590045

**Published:** 2020-10-20

**Authors:** Timothy E. Hoffman, William H. Hanneman, Julie A. Moreno

**Affiliations:** Department of Environmental and Radiological Health Sciences, College of Veterinary Medicine and Biomedical Sciences, Colorado State University, Fort Collins, CO, United States

**Keywords:** aging, systems biology, agent-based modeling, response signature, oxidative stress, tau, Alzheimer’s disease

## Abstract

Alzheimer’s disease (AD) is the leading cause of dementia and one of the most common causes of death worldwide. As an age-dependent multifactorial disease, the causative triggers of AD are rooted in spontaneous declines in cellular function and metabolic capacity with increases in protein stressors such as the tau protein. This multitude of age-related processes that cause neurons to change from healthy states to ones vulnerable to the damage seen in AD are difficult to simultaneously investigate and even more difficult to quantify. Here we aimed to diminish these gaps in our understanding of neuronal vulnerability in AD development by using simulation methods to theoretically quantify an array of cellular stress responses and signaling molecules. This temporally-descriptive molecular signature was produced using a novel multimethod simulation approach pioneered by our laboratory for biological research; this methodology combines hierarchical agent-based processes and continuous equation-based modeling in the same interface, all while maintaining intrinsic distributions that emulate natural biological stochasticity. The molecular signature was validated for a normal organismal aging trajectory using experimental longitudinal data from *Caenorhabditis elegans* and rodent studies. In addition, we have further predicted this aging molecular signature for cells impacted by the pathogenic tau protein, giving rise to distinct stress response conditions needed for cytoprotective aging. Interestingly, our simulation experiments showed that oxidative stress signaling (via *daf-16* and *skn-1* activities) does not substantially protect cells from all the early stressors of aging, but that it is essential in preventing a late-life degenerative cellular phenotype. Together, our simulation experiments aid in elucidating neurodegenerative triggers in the onset of AD for different genetic conditions. The long-term goal of this work is to provide more detailed diagnostic and prognostic tools for AD development and progression, and to provide more comprehensive preventative measures for this disease.

## Introduction

Alzheimer’s disease (AD) has become the most prevalent age-dependent neurodegenerative disorder, with over 46.8 million reported cases of AD worldwide (Cummings et al., 2016). If disease diagnostics and treatments remain in their current ineffective state, this metric is expected to at least triple by 2050, meaning 1 in 85 individuals worldwide will be living with the disease (Brookmeyer et al., 2007). The United States alone is expected to see > 60% increases in AD cases by 2025 (Weuve et al., 2015). AD and similar age-dependent neurodegenerative diseases are characterized by severe memory impairment and widespread loss of brain function, with AD predominantly affecting neurons of the hippocampus (Moodley and Chan, 2014; Milenkovic et al., 2014). At the cellular level, neurodegeneration is the cumulative result of oxidative stress, tau accumulation and misfolded protein stress, inflammation, loss of mitochondrial function, impaired autophagy processes, and more (Kraemer et al., 2006; Hekimi et al., 2011; Menzies et al., 2015; Wang and Hekimi, 2015). While many cellular pathways and processes contribute to this neuronal damage, age is the strongest and most ubiquitous risk factor (Niccoli and Partridge, 2012; Guerreiro and Bras, 2015), warranting AD researchers to continually revisit the fundamentals of biological aging.

It is well understood that mitochondria play a critical role in the aging process (Hekimi et al., 2011; Wang and Hekimi, 2015; Labbadia et al., 2017; Hoffman et al., 2017, 2018). Because of this, energetically demanding cells (e.g., myocytes, neurons) that rely heavily on the integrity of their abundant mitochondrial populations are most affected by age. Age-dependent declines in mitochondrial function are heavily implicated in the pathogenesis of AD (Piaceri et al., 2012), leading researchers to target mitochondrial health through a variety of strategies in combatting AD onset and progression (Anand et al., 2014). The exacerbated mitochondrial dysfunction that ensues in symptomatic AD outcomes in humans can theoretically be ameliorated through (i) direct tau binding and reduced tau oligomerization, (ii) direct radical scavenging to curb mitochondrial ROS production, and (iii) targeting bioenergetic efficiency directly in an attempt to rescue failing neurons (Fatouros et al., 2012; Anand et al., 2014; Guerreiro and Bras, 2015). While these approaches are rooted in sound theory and have promising pre-clinical results, human studies have seen negligible efficacy (Anand et al., 2014). One particular pathway that has been gaining considerable attention for diseases of aging is the mitochondrial unfolded protein response (UPR^*mt*^), a response that is intended to protect mitochondria and is activated by a wide spectrum of stressors (Nargund et al., 2012). Analysis of the frontal cortex of human AD brains has shown significant increases in UPR^*mt*^ genes, demonstrating that this stress response is spontaneously activated in an attempt to improve mitochondrial content and combat the disease (Beck et al., 2016). Further pharmacological activation of the UPR^*mt*^ has been shown to enhance mitochondrial proteostasis and alleviate neurological dysfunction in nematode and mouse AD models (Regitz et al., 2016; Sorrentino et al., 2017).

Spontaneous mitochondrial dysfunction remains a target of investigation for neurodegeneration, but it is far from the only significant condition that renders neurons vulnerable to stress. Factors that impair neuronal proteostasis over time play an equally important role (Anisimova et al., 2018). It is well understood that AD rapidly affects the brain through the aggregation of aberrant β-amyloid (Aβ) peptides and tau proteins (Murphy and LeVine, 2010; Lopes et al., 2016; Butterfield and Boyd-Kimball, 2018), and properly balanced proteostasis pathways are essential to hinder the activity of these aggregates. Specifically, targeting and controlling the ER-based unfolded protein response (UPR^*ER*^) has resulted in substantial neurological protection and recovery in mammalian models of tauopathy (Radford et al., 2015; Halliday et al., 2017) and similar neurodegenerative models of aberrant prion protein propagation (Moreno and Mallucci, 2010; Moreno et al., 2012, 2013). The UPR^*ER*^, when dysregulated by Aβ and tau overexpressions, is also linked to deleterious mitochondrial deficits (Poirier et al., 2019); in the same regard, therapeutic pharmacological targeting of the UPR^*ER*^ has shown concomitant activation of the UPR^*mt*^ and overall improvement in AD phenotypes (Regitz et al., 2016). These observations highlight the complex interconnectivity between cellular proteostasis mechanisms and mitochondrial dynamics.

The adjacent pathways by which cells adapt to these mitochondrial, oxidative and proteotoxic insults are also paramount to their survival. Particular stress responses that appear to curb AD pathogenesis under the appropriate circumstances are macroautophagy, mitophagy (Menzies et al., 2015), and the Nrf2/SKN-1- and FoxO/DAF-16-mediated oxidative stress responses (Hesp et al., 2015; Johnson and Johnson, 2015). Autophagy processes, especially those concerning the mitochondria, have shown to have a critical impact on the way organisms age, with significant neurological deficits resulting from autophagic dysregulation (Palikaras and Tavernarakis, 2012; Palikaras et al., 2015; Hoffman et al., 2017). Alzheimer’s brains often display this level of dysregulation naturally, and many molecular and cognitive improvements have been achieved in mammalian AD models upon both autophagy and mitophagy hyperactivation (Du et al., 2017; Rocchi et al., 2017). With regard to the Nrf2/SKN-1 and FoxO/DAF-16 oxidative stress responses, probing the elements of these pathways have shown some decreases in age-dependent stress in a number of experimental models (Robida-Stubbs et al., 2012; Sun et al., 2017). Furthermore, tau-challenged neuronal models have naturally elicited these oxidative stress responses (Chew et al., 2015), and further pharmacological upregulation of either Nrf2/SKN-1 or FoxO/DAF-16 has ameliorated tau-mediated neurodegenerative disease phenotypes (Chen et al., 2015; Johnson and Johnson, 2015).

While all of these cellular events and responses are involved in the aging process and in neurodegenerative results, elucidating the finite relationships within this complex network is imperative. Examples of these relationships are the definitive links between autophagy and ER stress (Jo et al., 2014) and the links between mitochondrial integrity and proteostasis (Labbadia et al., 2017; Sorrentino et al., 2017). In addition to understanding the pathways involved and their connected relationships, it is also important to consider the temporal nuances of such cellular activities. Neuronal integrity at various life stages is rooted in specific quality control processes. Early-life maintenance requires functional oxidative stress responses to sustain an appropriate redox balance (Hekimi et al., 2011). Early-life maintenance also requires reliable mitochondrial stress responses to perpetually weed out faulty mitochondrial materials before they have the ability to propagate (Wang and Hekimi, 2015; Hoffman et al., 2017). Later in life, neurons have already accumulated the oxidative stressors and protein aggregates that cause damage; this is when the cell relies heavily on robust autophagy processes (Menzies et al., 2015) and potent stress responses (Kraemer et al., 2006; Halliday et al., 2017) intended to remove damaging materials and absorb the impacts of damaging biomolecules. These temporally nuanced cellular mechanisms highlight the complex nature of pharmacologically altering all of these processes to optimize neuronal function at all stages of life. These time-based and magnitude-based systems biology obstacles underscore the critical need for more comprehensive experimental and therapeutic approaches for AD research.

The idea that neuron subpopulations have selective vulnerability has been postulated to explain degeneration patterns within the brain (Bartsch and Wulff, 2015; Roselli and Caroni, 2015; Mattsson et al., 2016). More generally, types of response-based vulnerabilities have been theorized for any neuronal subpopulation, with emphasized examples involving UPR^*ER*^ functionality (Filézac de L’Etang et al., 2015). The vast network of general molecular conditions (e.g., conserved stress responses, redox environments) that precipitate and define vulnerability phenotypes within cellular populations are difficult to characterize with the limitations of conventional experimental approaches. An increasing number of researchers posit that integrative computational methods will provide more complete information for such queries (Mooney et al., 2016). Techniques that integrate *in silico* methods with *in vitro* and *in vivo* experiments are now frequently emerging for neurodegenerative research (Wood et al., 2015; Hassan et al., 2018). Previous *in silico* approaches of this nature have been fruitful in theorizing oxidative characteristics of aging and disease (Cloutier and Wellstead, 2012), lipid raft characteristics in AD development (Santos et al., 2016), and metabolic shifts in normal aging (Hastings et al., 2019).

We have previously established a multimethod computational simulation approach that has demonstrated the ability to characterize relationships between aging and mitochondrial pathways (Hoffman et al., 2017). In this study, we have built upon our foundational studies to construct a computational simulation tool that integrates all of the aforementioned molecular mechanisms characterizing neuronal conditions of neuronal vulnerability. The design of this simulated network relates to experimental observations within the model organism *Caenorhabditis elegans*, which has been a useful model in the field (Alexander et al., 2014). Such conditions are recognized here as the measured set of pathways and responses that drive and accompany the aging process. This set of measurements produced by the simulation can be considered a time- and magnitude-based *molecular signature*, similar to signatures previously developed and used for toxicological screening assays (Simmons et al., 2009) and cancer cell prognostic studies (Lal et al., 2017). The signature outputs presented here vary within the cellular population and defines phenotypic outcomes of resilience or vulnerability, based on the experimentally determined pathway relationships. Analyzing this molecular signature over the lifespan of an organism is the hallmark of this approach. To prevent neuronal vulnerability to aging and protein misfolding, our previous work suggests that appropriate cellular activities of the UPR^*mt*^ and UPR^*ER*^ stress responses are critical (Halliday et al., 2017; Hoffman et al., 2017). Here the simulation outputs fall in line with these hypotheses and predict temporal trajectories that result in neuronal populations most vulnerable to oxidative and proteotoxic stressors. Overall, this approach provides the field with a means to more accurately grasp (1) the timing of multiple stress events in AD development, and (2) the neuroprotective effects of stress response mechanisms in AD prevention and treatment.

## Results and Discussion

### Simulation Design and the Molecular Signature

**FIGURE 1:**
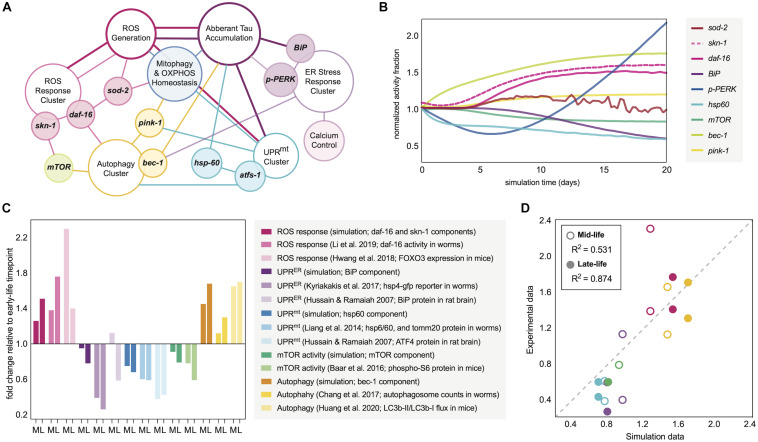
Molecular network design and simulation outputs. **(A)** Age-dependent oxidative and proteomic stressors defined in the aging response network of interest. The model is designed with reactive oxygen species (ROS) as the epicenter of spontaneous time-dependent stress, which can drive protein accumulation and a profile of stress response clusters. Such clusters (defined in the schematic by open ellipses) are driven by critical regulators (filled-ellipse genes overlapping with clusters) and downstream targets and functions (filled ellipses adjacently connected to clusters). The connections defined in the schematic define which nodes have influence on others, with the color of the connection defining the origin and thus direction of the effect. The computational definitions of these positive and negative regulations within the molecular network are defined in the Supplementary Information. **(B)** Temporal trajectory of the molecular response signature in normal aging nematode neurons. The fold-change in the response is normalized to the 0-day mark, assumed to be the first day of nematode adulthood. **(C)** Average fold-change in response indicators seen experimentally in wild-type organisms as they relate to the simulated aging signature. Mid-life (M) and late-life (L) values are plotted for each relative to early-life activity values. The simulation components plotted are for day 6 (M) and day 11 (L) values relative to those at day 0. Experimental data from nematodes represents day 4–6 (M) and day 8–11 (L) values relative to those at the first day of adulthood. Experimental data from rats represents 1–6 month (M) and 18 month (L) values relative to those within the first month of life. Experimental data from mice represents 6–18 month (M) and 18–30 month (L) values relative to those within the first 3–6 months of life, where consistency allowed. **(D)** Correlation analysis between the simulation outputs and experimental data collected in figure panel 1C. Plotted values are grouped by mid-life and late-life definitions, and are colored based on the stress response grouping from panel 1C. Dotted line represents ideal 1:1 correlation trajectory, and actual *R*^2^ calculations are displayed in the legend.

The simulation constructs used in this study centered around age-dependent cellular and mitochondrial oxidative signaling, similar to our previous work, however the model presented here was designed with a wider array of stress responses experimentally determined to be significant to neuronal vulnerability phenotypes. The nodes producing the normalized expressions of critical markers and stress response activities are shown as functional clusters in Figure 1A. This network was constructed within our simulation interface in part by integrating the on-off behavior of Boolean network modeling, however, the nodes were designed using system-dynamics techniques (ODE-based) of primarily saturable Hill functions, which we have previously modeled for nuclear receptors (Hoffman et al., 2018). This allowed us to produce normalized response fractions for each of the molecular markers involved in the full signature of interest. This conversion to an equation-based network also allowed us to integrate the response nodes with an agent-based system of (1) varying cellular behavior within a cell population (2) varying mitochondrial populations within each cell, (3) crosstalk between the molecular network and cellular and mitochondrial behaviors, and (4) nuanced relationships between the molecular network and cellular agent phenotypic outcomes. Expressions concerning mitochondrial conditions and behavior are delineated in Box S1 and Supplementary Figure S1, whereas the expressions concerning the cellular biomolecular network and agent population behaviors are delineated in Boxes S2, S3, and Supplementary Figure S2; all corresponding model parameters are tabulated in Supplementary Table S1.

The initial simulation output was determined to be the molecular signature of 9 dynamic nodes of interest as they correspond to markers within *C. elegans*: *hsp-60* (UPR^*mt*^ activity), *BiP* (total UPR^*ER*^ activity), *p-PERK* (translational branch of the UPR^*ER*^), *skn-1* activity, *daf-16* activity, *sod-2* (mitochondrial superoxide dismutase activity), *bec-1* (general macroautophagy machinery), *mTOR* (anti-autophagic activity), and *pink-1* (pro-mitophagy activity). The patterns of response fractions for each of these functional pathways were measured in the simulation over a 20-day period (the higher end of lifespan probability for a wild-type nematode; Stroustrup et al., 2016). This temporal trajectory is shown in Figure 1B, with distinct patterns of stress response dysregulation upon normal aging.

To validate the molecular changes with age that we observed in our simulation, we performed a systematic search for longitudinal molecular datasets of the same response processes. Although such longitudinal studies are scarce, there are several that make quantitative conclusions either with the direct protein target of interest, with an ortholog for species translation, or with a signaling molecule as a process proxy. Making direct comparisons of our molecular changes to those seen in aging organisms, we find a striking number of similarities (Figure 1C) and a high quantitative correlation (Figure 1D). Predictions of the model correlated with mid-life and late-life changes in biological activities, but the model produced more accurate predictions for late-life activity, which is more favorable for making neurodegenerative inferences.

In comparing the simulation results with experimental findings, several examples were noteworthy. The simulation predicts an age-dependent dysregulation of autophagic processes, which has manifested experimentally in aging nematodes as an accumulation of autophagosome content in neuronal tissue with variable changes in autophagic flux (Chapin et al., 2015; Chang et al., 2017; Huang et al., 2020). Also in accordance with preliminary simulation results, the spontaneous nuclear localization of *daf-16* along with *skn-1* expression have been shown to increase in normal aging nematodes (Hwang et al., 2018; Li et al., 2019). It is important to note that skn-1/Nrf2 activity across nematode and mammalian lifespans has been measured in variable expression patterns (Zhang et al., 2015), and even when increased, skn-1/Nrf2 activation alone has not demonstrated enough compensatory effects to combat the natural oxidative stressors of aging. The simulation results also mirror experimentally observed impairments in UPR^*ER*^, UPR^*mt*^ and mitochondrial maintenance with neuronal age, allowing permissive oxidative damage and vulnerability to proteotoxic insults (Yasuda et al., 2006; Hussain and Ramaiah, 2007; Taylor and Dillin, 2013; Liang et al., 2014; Labbadia and Morimoto, 2015; Morsci et al., 2016; Frakes and Dillin, 2017; Kyriakakis et al., 2017). Since the UPR^*mt*^ is still incompletely characterized in mammalian cells, we have used ATF4 levels as a proxy for the mammalian response in our validation since this transcription factor is activated upon general mitochondrial stress in mammals alongside the typical integrated stress response (Guo et al., 2020), and indeed ATF4 protein levels decline with age (Hussain and Ramaiah, 2007). Additionally, mTOR activity declines are observed in our simulation and experimentally (Baar et al., 2016).

### Nodes Triggering Vulnerability Phenotypes

**FIGURE 2:**
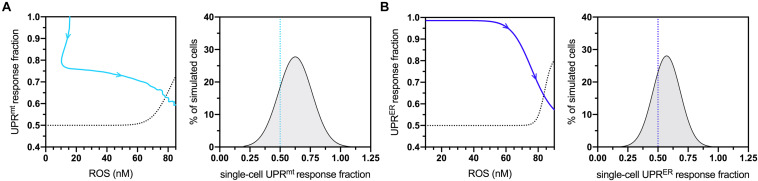
Cellular vulnerability characterizations of UPR^*mt*^ and UPRER activities with respect to increasing age-dependent oxidative insults. **(A)** Dose-response curve denoting bistable UPR^*mt*^ activity in response to age-dependent ROS increases, with the UPR^*mt*^ activity of late-life cell aggregates approaching 0.5 (left panel) The blue line shows the simulated time-dependent change in this relationship, where aging increases in ROS cause a decline in UPR^*mt*^ activity; the dashed line is fitted to a scenario where a fraction of ROS levels are artificially removed toward the end of life, demonstrating a potentially irreversible bifurcation into a new vulnerable state characterized by 50% loss of UPR^*mt*^ capacity. Also shown is the UPR^*mt*^ activity distribution of simulated cells at the 20-day mark, with the lower end (26%) satisfying the <0.50 condition. **(B)** Dose-response curve denoting bistable UPRER activity in response to age-dependent ROS changes, with the UPRER activity of late-life cell aggregates approaching 0.5 in normal aging. Also shown is the UPRER activity distribution of simulated cells at the 20-day mark, with the lower end (32%) satisfying the <0.50 condition.

To determine sensitive controllers of the simulated network, a standard global sensitivity analysis was performed for all static parameters. The resulting sensitivity coefficients are displayed in Supplementary Table S2, with expected significant sensitivity found at all stages of life for the parameters defining age-dependent ROS production and elimination. The most sensitive values falling balow the level of strong sensitivity corresponded to the coefficients returned for UPR^*mt*^- UPR^*ER*^- and skn-1-based activities, suggesting that they also play a critical role in determining changes within the molecular system at both mid- and late-life stages. Due to these formal observations, both UPR^*mt*^ and UPR^*ER*^ activities were analyzed with respect to the model’s natural age-dependent increase in ROS levels. The temporal movement through these dose-response plots are displayed in Figures 2A,B (left panels) for normalized UPR^*mt*^ and UPR^*ER*^ activities, respectively. The temporal movement displayed impairments in both these responses, and removal of ROS content at late-life stages did not recover these impairments; instead, the model predicted that these cells would continue a trajectory within the dose-response that approaches 50% activity levels for both responses. Because of these data, these activity values were chosen as triggering conditions for cellular vulnerability within the simulated cell population. The population distribution of UPR^*mt*^ and UPR^*ER*^ activities at the end of life (day 20) are displayed in Figures 2A,B (right panels), with over a quarter of the cells satisfying these impaired conditions. These data are consistent with findings detailing defective proteostasis mechanisms triggering cellular vulnerability phenotypes in neurodegenerative disorders (Filézac de L’Etang et al., 2015).

**FIGURE 3:**
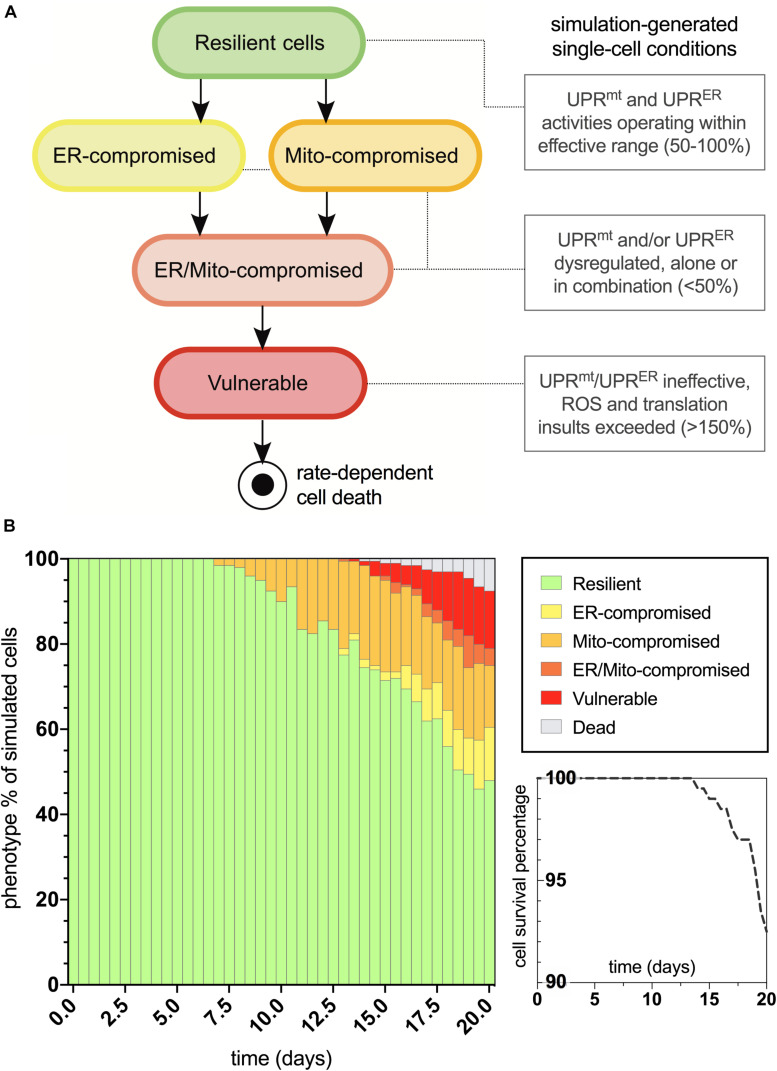
Bifurcated phenotypic characterizations and simulation results for normal aging nematode neurons. **(A)** States of increasing cellular vulnerability with respect to the UPR^*mt*^ and UPR^*ER*^ analyses, which result in an ultimate vulnerability state conditionally bound by age-dependent oxidative and proteotoxic insults. **(B)** Simulation results of cellular phenotype percentages over time for normal aging neurons, including theorized rate-dependent cell survival with respect to vulnerability increases.

The age-dependent cellular conditions defined in the computational network were then used to produce bifurcations through phenotypic states of vulnerability that were quantified for the simulated cell population (Figure 3A). At simulation startup, all cell agents exist with the fully resilient phenotype and no deviations from the normalized response activities. Cells then conditionally transition to states of UPR^*mt*^ and/or UPR^*ER*^ compromised activities, and when both mitochondrial and ER deficiencies are satisfied, the cells are then rendered vulnerable to age-dependent increases in oxidative and proteotoxic stressors. Once cells enter into the final vulnerability state, they then move through a rate-dependent transition into cell death, where they are finally removed from the simulation. After designing this bifurcation landscape, the phenotype percentages for normal aging were quantified (Figure 3B). As we have predicted and discussed in our previous work, compromised mitochondrial processes developed early on in aging, with ER deficits occurring later in life. Ultimately, a steady increase in full vulnerability was observed shortly after day 12, with a gradual decline in cell survival seen after day 14 (5% lost at day 19). In a direct extrapolation of these simulated nematode values to a human lifespan of 100 years, vulnerable neurons would surface around 60 years with a 5% neurodegenerative loss occurring at 95 years. While such a linear extrapolation for organismal lifespan has not been well studied, this type of analysis appeared suitable for our study inferences of normal and tau-exacerbated aging.

### Neuronal Vulnerability Quickens With Increased Tau Aggregation and Oxidative Signaling

**FIGURE 4:**
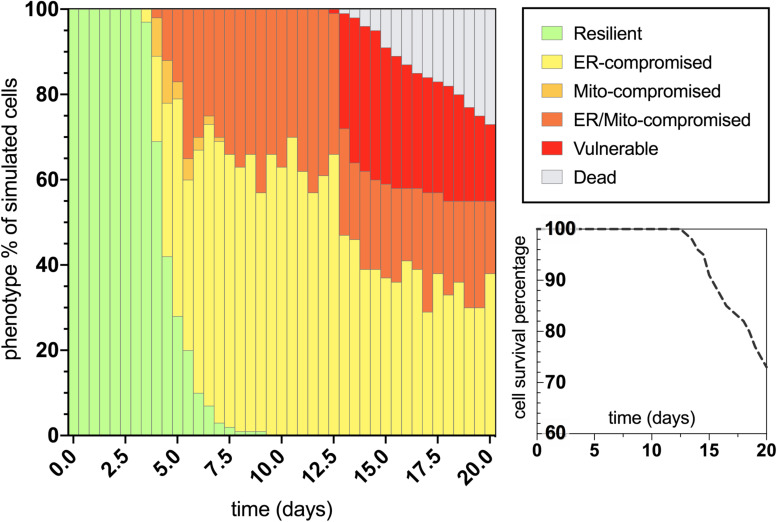
Bifurcated phenotypic characterizations and simulation results for tau-exacerbated nematode neurons. Simulation results of cellular phenotype percentages over time for tau-insulted (100% increase) aging neurons, including theorized rate-dependent cell survival with respect to vulnerability increases.

After characterizing the molecular signature in the trajectory of normal aging, we then applied an assumed 100% increase in tau aggregation within the simulation interface to quantitate its effects on the predicted outputs. The bifurcated phenotypic characterizations that resulted after integrating this tau insult are displayed in Figure 4. The maintenance of the resilient neuronal phenotype is markedly decreased, with absolutely no cell agents remaining in this state after 9 days of age (theoretically translates to age 45 for outstanding tau-insulted humans). Meanwhile, 5% degenerative loss occurred in the simulation at day 14 (theoretically translates to age 70 for outstanding tau-insulted humans, consistent with the typical age of neurodegenerative symptom onset). As expected, ER stress is substantially heightened in the tau-exacerbated simulation and UPR^*ER*^ dysregulation precedes or coincides with mitochondrial deficits, in contrast to the normal aging results. Cell survival was markedly decreased in the tau-exacerbated network, emulating the neuronal dysfunction and neurodegenerative outcomes seen in tau-expressing pro-aggregating nematodes (Fatouros et al., 2012). Although this computational system was used in the current study to only look at two discrete states of non-aggregating and pro-aggregating tau, we encourage future works to simulate graded levels of tau and identify nuances to tauopathy onset and progression.

**FIGURE 5:**
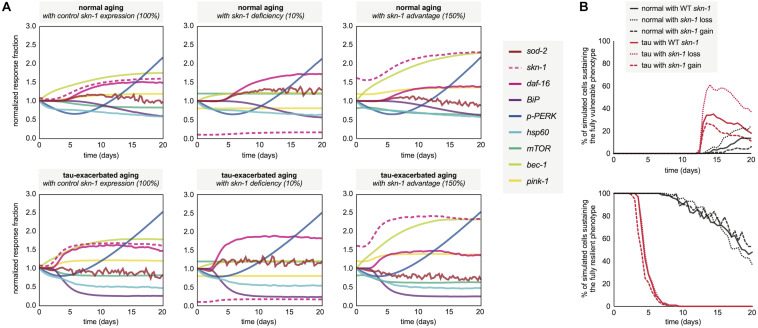
Molecular signatures in normal and tau-exacerbated aging neurons sustaining variable activities of the *skn-1*-mediated oxidative stress response. **(A)** Changes in the temporal trajectory of the simulation-generated molecular signature are shown for normal aging neurons (top panels) and for tau-exacerbated neurons (bottom panels) after partial skn-1 ablation and skn-1 overexpression. Reduction and overexpression percentages (10 and 150%, respectively) were chosen based on average experimental efficiencies of such approaches to make the simulation outputs presented here feasible and translatable in future experimental settings. **(B)** Varying time-course phenotype measurements for each of the six genetically-altered groups. The age-dependent genesis of the fully vulnerable phenotype with corresponding cell percentages are shown (top panel) as well as the maintenance of the most resilient phenotype and its corresponding cell percentages (bottom panel). For reference, the most resilient state and the fully vulnerable state are displayed in Figure 3B in green and red, respectively.

Following the neuronal phenotyping analyses for normal and tau-exacerbated aging, the molecular signatures for both scenarios were generated by the simulation to observe differences in the response trajectories of both models (Figure 5A, left panels). In addition, because of *skn-1* sensitive system control revealed during the development of the computational network, the expression of this node was altered by assumption-based percentages for additional experiments to gain insight about loss (Figure 5A, middle panels) or gain (Figure 5A, right panels) of function in *skn-1*-mediated activities for both normal and tau-exacerbated aging. This genetic alteration was also chosen as many therapeutic approaches have been successful in targeting and modulating the *skn-1/Nrf2* antioxidant response pathway (Johnson and Johnson, 2015). Simulation results indicate that *skn-1* alterations resulted in slight alterations in maintaining ER and mitochondrial functionality within the molecular signature, particularly in autophagy and mitophagy activities. The changes in skn-1 led to more substantial benefits or consequences in mitigating oxidative damage, especially in the tau-exacerbated worms. These *skn-1*-dependent changes in the molecular signature trajectories corresponded with changes in age-dependent vulnerability phenotyping, displayed in Figure 5B. Simulation results indicate that *skn-1* was not required for the maintenance of the most resilient phenotype in either natural or tau-exacerbated aging (bottom panel), however, it was exceptionally important in mitigating chronic oxidative stress required for simulated cell agents to enter into the final vulnerability phenotype (top panel) that is proportional with poor cell survival. These simulation data are consistent with experimentally observed relationships regarding *skn-1/Nrf2*-mediated cytoprotection in the context of antioxidant functions, autophagic maintenance and tauopathy amelioration in worms, mice and humans (Chew et al., 2015; Rojo et al., 2017; Tang et al., 2017). More specifically, a ∼25% reduction in hippocampal mRNA of autophagy regulators occurs in aging *Nrf2*^–/–^ mice compared to WT (Tang et al., 2017), which is on the same order of autophagy reduction we see in our simulation after *skn-1* ablation (Figure 5A). This systemic collapse of proteostasis emphasizes the importance of *skn-1/Nrf2* in preventing tauopathy-driven death.

### Neuronal Survival During Aging Depends on Multiple Stress Response Pathways

**FIGURE 6:**
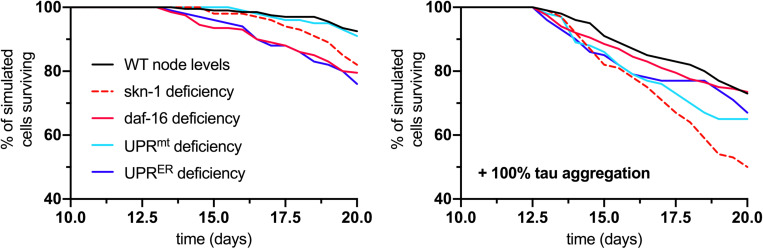
Analysis of cell survival in normal aging (left panel) and tau-exacerbated aging (right panel) neurons with respect to different node deficiencies. Time-course plots display no change in genetic nodes (denoted WT), and reduced levels of several nodes and response activities. Nodes of interest that were altered include skn-1, daf-16, UPR^*mt*^, and UPR^*ER*^ activities, as these were selected as having strong determining potential against the phenotypic outputs and ultimate neuronal vulnerability.

As a final output of the simulation, neuronal survival was assessed over the full 20-day lifespan analysis for normal and tau-exacerbated cell populations and for response deficiencies. The results displayed here compare cell survival rates between the fully functional response network and groups lacking *skn-1*, *daf-16*, UPR^*mt*^, or UPR^*ER*^ activities (Figure 6). For normal aging (left panel), all simulated deficiencies except for the UPR^*mt*^ caused a decrease in cell survival. This is consistent with finding that UPR^*mt*^ modifications in normal aging nematodes without stress led to no stark changes in longevity (Nargund et al., 2012; Bennett et al., 2014). For tau-exacerbated aging (right panel), all simulated deficiencies, including the UPR^*mt*^, caused a decrease in cell survival, suggesting all of these pathways are critical in mitigating tau-dependent damage in aging nervous systems. This is especially consistent with findings that display the profound ameliorative effects of daf-16/FOXO activation, specific UPR^*ER*^ activation and regulation, UPR^*mt*^ activation and mitophagy on proteotoxic AD models of neurodegeneration (Chen et al., 2015; Marcora et al., 2017; Sorrentino et al., 2017; Fang et al., 2019). It is speculated here that all of these responses must act in concert to maintain resilient neuronal phenotypes, but the age-dependent deterioration of these quality control processes is yet to be fully understood.

### Investigating and Targeting Comprehensive Pathway Maintenance During Aging

Because developing AD appears to be a highly probable event in the aging process and due to the refractory nature of the associated pathophysiology, actions taken toward preventing the triggers of AD are currently the most suitable options (Guerreiro and Bras, 2015; Moffitt et al., 2016). Such actions are typically environmental interventions intended to reduce AD risk factors. Environmental enhancements include cognitive training, stress management, social engagement, physical activity, lipoprotein control for vascular health, nutritional approaches and phytochemical supplementation (Han and Han, 2014). Certain stilbenoid phytochemicals such as resveratrol and pterostilbene have gained popularity for their ability to prime cells to mount beneficial responses and combat age-dependent damage (Anand et al., 2014; Wang and Hekimi, 2015; Hoffman et al., 2017). All of these preventative measures appear to have selective cellular benefits against neuronal dysfunction that appears to silently progress for many years before pathological AD onset (Huang and Mucke, 2012). Any neurodegenerative disease, as we have quantitatively studied in this project for tau-related contexts, appears to be born out of a multitude of intrinsic and environmental factors (Huang and Mucke, 2012; Serý et al., 2013; Xie et al., 2014), and thus it is likely it will take multiple environmentally based interventions to have a fully protective or even restorative effect on all neuronal populations.

Investigating all of these critical conditions that render neuron populations vulnerable to the triggers of AD and similar neurodegenerative diseases is difficult to perform *in vivo*. Computational models of aging have provided many insights into the systems biology obstacles of researching AD (Mooney et al., 2016), and our previous simulation approach has offered value to the field of age-related diseases (Hoffman et al., 2017). However, this previous model was not without its limitations. Primarily, the previous simulation (1) lacked nuanced details in the phenotypes describing age-dependent vulnerability, (2) would have benefited from more translational power in experimental biomarkers specific to genetic declines in disease states, (3) and warranted more robustness and usability as a computationally intensive model. In the current study, we aimed to remedy these shortcomings of the previous model. The simulation presented here relies on a defined network capable of revealing heavily nuanced vulnerability phenotypes in aging organisms. The simulation was also constructed in a manner that allows AD researchers to follow up on defined reporter genes and biomolecules of stress responses highly relevant to aging and to the disease progression; such genes have been well-defined for a simple model organism. Finally, the simulation was mathematically condensed in areas that did not change critical network motifs for this study, and it was also expanded upon in areas that required more detail for aging and AD specifics; this ultimately allowed for a less computationally intense model to increase agent-based population robustness and user friendliness. While we believe the current model is the most useful iteration yet, future expansions including other gene sets and processes will lead to even more promising findings; these include the growing information on neuroinflammatory responses in aging (Talwar et al., 2019; Falcicchia et al., 2020), cellular crosstalk within the aging brain and the functional decline in the astrocyte population (Han et al., 2020), and newly uncovered aging dynamics involving a DNA damage response (Zheng et al., 2020). These current and future modeling improvements will bring us to better investigative tools for integrative experimental and computational neurodegenerative research.

## Conclusion

In conclusion, the simulation results generated here highlight the importance of response pathway maintenance in degenerative aging and AD development, as evidenced by the temporal molecular signatures predicted. Of importance, this study quantitatively corroborates the growing understanding that mitochondrial and ER maintenance through specific UPR^*mt*^ and UPR^*ER*^ programming play a critical role in mitigating age-dependent vulnerability to proteotoxic and oxidative stressors. Additionally, this work confirms the significance of oxidative stress responses in prolonging age-dependent cellular degeneration. Interestingly, our simulation experiments showed that oxidative stress signaling (via *daf-16* and *skn-1* activities) does not substantially protect cells from all the early stressors of aging, but that it plays an essential role in preventing a late-life degenerative cellular phenotype. As comprehensive studies integrating these responses continue to surface, the scientific community will be able to develop more elaborate prognostic tools for age-related diseases and preventative treatments.

## Materials and Methods

### Hierarchical Simulation Design and Agent-Based Modeling

In order to simulate the mitochondrial and cellular relationships of interest (Figure 1A), we formulated our model using AnyLogic^®^ multimethod simulation software (version 8.3; The AnyLogic Company, Chicago, IL), which allowed us to develop a hierarchical agent-based simulation scheme, as we have previously described (Hoffman et al., 2017). Access to the simulation is described in the subsequent section regarding data availability. To briefly describe the methodology, this simulation was constructed with a population of cell agents, with each individual cell agent containing its own population of mitochondrion agents. Each agent contained discrete event statements and system-dynamics reaction networks, both of which were programmed to deal with natural biological stochasticity. Cellular agents contained their own continuous genetic and biochemical networks, which produced an array of intracellular conditions, referred to here as the molecular signature. These conditions were analyzed for sensitivity and determining control over the system to reveal phenotypes of response deficiencies and ultimately vulnerability, which are highlighted in the results section. These bifurcating transitions that allow agents to move through different states are written as java-based expressions and are all unpacked in the Supplementary Information.

### Deterministic Modeling Components

As Boolean approaches have been successful in representing mathematical relationships between biomolecules and genes, especially for systems of aging (Mooney et al., 2016; Verlingue et al., 2016; Schwab et al., 2017), this discrete binary concept was converted to Hill-based deterministic (ODE-based) relationships of gene expressions and response activities to produce a continuous computational simulation that could be easily managed temporally alongside biochemical equation frameworks and the aforementioned agent-based phenotyping methods. The manually-selected and organized network is hinged primarily upon age-dependent oxidative signaling and many adjacent stress responses, as depicted in the results section. These curated molecular relationships are detailed in Figure 1A, and all of the equations and corresponding parameters used are organized, tabulated and presented in the Supplementary Information.

### Sensitivity Analysis

To determine the quantitative significance of certain parameters and functions within the system, a similar global sensitivity strategy was used that has been described previously (Tam et al., 2015; Hoffman et al., 2017). Briefly, each parameter was individually reduced by 5%, and the resulting model output of cellular ROS levels was assessed. This was done for all static parameters within the model, and the response variable changes were measured at particular time points determined for young age (day 5), midlife (day 10), and old age (day 19). Sensitivity coefficients (SC) were calculated as:

$$S\,C=\left(\frac{\partial O_{i}}{\partial P_{j}}\right)\times \left(\frac{P_{j}}{O_{i}}\right)=\left(\frac{\partial O_{i}}{O_{i}}\right)\times 20$$

where *SC* represents the normalized sensitivity coefficient, *O*_*i*_ is the model output value, *P*_*j*_ is the value of the parameter of interest, and ∂ represents the partial derivative of either the parameter value or model output. When |SC| ≥ 1.0000, i.e., when the proportional change in the response variable is greater than the proportional change in the parameter, the parameter is deemed to have significant sensitive control over the respective output at the specified age. SC absolute values falling between 0.1000 and 0.9999 were also considered for further analyses of system control.

### Mathematical Analyses and Graphical Visualizations

Statistical analyses of Gaussian distributions, curve-fitting, correlational graphing, and survival analyses were performed using GraphPad Prism software (version 8). Illustrations and vector drawings were created using ChemDraw Professional (version 15.1) and Adobe Illustrator.

## Data Availability Statement

The simulation tool is available as both a java applet and downloadable file (.alp) via the AnyLogic Cloud (https://cloud.anylogic.com/model/ec37d0d4-fcbf-4158-9f31-d0a2e213dcb0). For use of the original file, users may download a free version (Personal Learning Edition) of the AnyLogic software from the company’s website. Other datasets upon which the results presented in the article are based can be requested by sending an email to the corresponding author.

## Author Contributions

TH, JM, and WH conceived the study ideas and theories, analyzed the data, and contributed to the preparation of this manuscript. TH constructed the computational tools and performed simulation experiments and developed the final figures. WH provided experimental tools and resources. All authors contributed to the article and approved the submitted version.

## Conflict of Interest

The authors declare that the research was conducted in the absence of any commercial or financial relationships that could be construed as a potential conflict of interest.

## Dedication

The final form of this manuscript is dedicated to the late Dr. William Hanneman, who always challenged us to be innovative scientists and deeply inspired us to be good people.
