# Supplementary material for: Network Simulations Reveal Molecular Signatures of Vulnerability to Age-Dependent Stress and Tau Accumulation
Source: Front Mol Biosci. 2020 Oct 20;7:590045. doi: 10.3389/fmolb.2020.590045 (PMC7606936; doi:10.3389/fmolb.2020.590045)
Supplement: Supplementary file 1 [file Data_Sheet_1.pdf]

Supplementary Material

Network Simulations Reveal Molecular Signatures of Vulnerability to Age-dependent Stress and Tau Accumulation

Timothy E. Hoffman, William H. Hanneman, and Julie A. Moreno†

Department of Environmental and Radiological Health Sciences, College of Veterinary Medicine and Biomedical Sciences, Colorado State University, Fort Collins, CO, USA

† **Correspondence:** Julie A. Moreno, julie.moreno@colostate.edu

SUPPLEMENTARY METHODS

1. General model design and time units for lifespan analyses

Upon model setup, the *C. elegans* lifespan amount was assumed to be 20 days (higher end of *C. elegans* age probability at 20 °C as per Stroustrup et al., 2016), and for temporal extrapolation calculations, human lifespan amount was assumed at 100 years or 29,200 days for simulation compatibility (approaching the maximum human lifespan, per Dong et al., 2016). Mitochondrial counts within each cell were taken from the Poisson distributions determined in our previous study (Hoffman et al., 2017). In each of the simulation experiments, 200 neurons were simulated to achieve robustness and user-friendliness in the distributions of node output and resulting phenotype analyses. This agent population was determined by iterative Monte-Carlo sampling to achieve reproducible results from each run of the intrinsically stochastic system.

2. Mitochondrial system-dynamics and discrete computational methods for normal aging

The framework for mitochondrial ROS generation, a pivotal player in the degenerative aging process, was condensed from our previous study (Hoffman et al., 2017) to focus more on the cellular genetic network of interest and more nuanced states of neuronal vulnerability for cellular phenotyping. Nodes that were condensed involved computational details of OXPHOS activity, mtDNA damage and intramitochondrial proteostasis mechanisms. These components have instead been inferred in other areas of the model: OXPHOS activity and mtDNA damage contributes to mtROS generation, and such constants account for this inference; mitochondrial proteostasis dictates global cellular protein health, and such constants determining mitochondrial stress responses and autophagic protein maintenance account for this dynamic intramitochondrial process. Simplification made to reach the current system did not significantly alter the behavior of the cellular environment, as the hierarchical modeling structure from the previous simulation

remains intact here, but instead this reduced the ability to simulate the same nuances in mitochondrial stress states as before. Since intermitochondrial characterizations were outside the scope of this study, this simplification was appropriate for producing a more applicable and easily understood tool that was less computationally intense for the community. The stochastic equations, agent transitions, and event statements pertaining to mitochondrion-level biochemical kinetics are displayed in Box S1, with corresponding descriptions and references of the logical derivations. The AnyLogic simulation schematics of the mitochondrial framework are also displayed for both the agent-based methods (Figure S1A) and deterministic ODE methods (Figure S1B).

Box S1: Equations, agent transitions, and event statements pertaining to mitochondrion-level biochemical kinetics.

Differential equations governing time-dependent mitochondrial ROS accumulation and elimination, comprised of general production and elimination constant distributions that resulted from our previous work (Hoffman et al., 2017) as well as logically-derived expressions for tau-mediated ROS accumulation and UPR^{mt}-mediated elimination (Cenini and Voos, 2016; Fiorese and Haynes, 2017; Kaufman et al., 2017; Pharaoh et al., 2016; Shpilka and Haynes, 2018). Expressions shown in purple are dictated from the cell-level environment:

$$\frac{d[ROS_{mt}]}{dt} = \frac{nM\ ROS_{mt}}{day} = K2 * \frac{Tau_{Agg}}{Tau_{Agg} + TM} - kMRE[ROS_{mt}] * UPRmt\_activity$$

Discrete events governing (i) movement through mitochondrial health and stress states and (ii) mitochondrial biogenesis and mitophagy events dictated by cellular activities of mitochondria-altering proteins:

Stochastic condition defining gain of mitochondrial ROS triggering a defective organelle phenotype:

$$[ROS_{mt}] > (triangular(30\ nM, 40\ nM, 50\ nM))$$

Static condition defining remediation of mitochondrial ROS restoring a healthy organelle phenotype:

$$[ROS_{mt}] < cell'sspecific\ threshold\ distribution\ value\ from\ previous\ expression$$

PINK-1-dependent rate of mitochondrial loss via mitophagy (agent removal):

$$\frac{Compromised\_mito\ destroyed}{day} = \frac{1}{pink1\_activity}$$

Basal and SKN-1-dependent rate of mitochondrial biogenesis (new agent addition):

$$days\ until\ agent\ addition = uniform(1,2) - \frac{1}{4} skn1\_activity$$

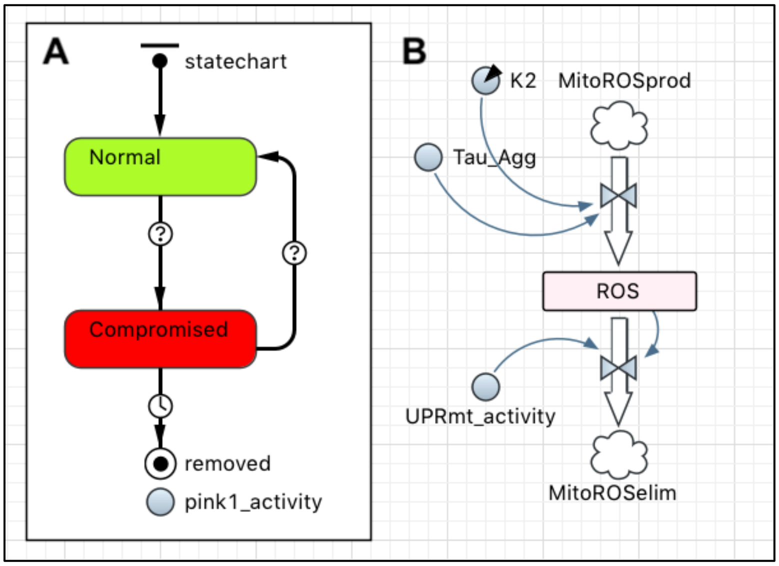

Figure S1. Simulation schematic displaying mitochondrial framework in the simulation software. **(A)** Agent state chart delineating stress-response-based conditional mitochondrial integrity. Normal and compromised agent states are dictated conditionally by mitochondrial stress variables. Final state ("removed") denotes mitochondrial removal via mitophagy, dictated by cellular *pink-1* expression and activity, shown as a dynamic variable in the model. **(B)** Simplified mitochondrial equation-based dynamics based primarily off of mitochondrial ROS production and content. Traditional stock-and-flow components are used to construct this system, where the box displays the intramitochondrial ROS concentration subject to dynamic changes, and the box arrows display flow rates to and from the ROS compartment, which are driven both by proteotoxic aggregation and stress response activities from the whole-cell level. Filled circles represent dynamic variables, whereas circles containing a black triangle represent static operational coefficients.

3. Cell-level system-dynamics and discrete-event computational methods for normal aging

Since the primary focus of this simulation study was to garner more nuanced information about neuronal vulnerability that arises in the aging process, the cellular dynamics were more intricate than those that make up the mitochondrial framework. The gene network of interest that is driven in part by age-dependent oxidative signaling is connected by deterministic nodes of activity as well as some biochemical reaction schemes describing protein accumulation and phosphorylation events. The nodes of activity for the genes and responses of the network were given deterministic ODE-based expressions of primarily Hill functions, in order to draw some similarities to the binary nature of Boolean gene networks that have been successful in previous studies (Schwab et al., 2017; Verlingue et al., 2016). This conversion allowed for (i) more graded activity to be seen within individual nodes for more detailed analyses, (ii) better implication of stochasticity within the cell agent population, and (iii) easier integration with other ODE-based frameworks and agent-based frameworks within the complete hierarchical model. The parameters and corresponding equations delineating this curated network are displayed in Table S1 and Box S2, respectively; the corresponding schematic of this equation network is shown in Figure S2A. The agent-based transitional events that allow cell agents in the simulation to possess different conditionally-bound phenotypes are expressed in Box S3; the corresponding schematic of this agent state chart is shown in Figure S2B.

**Table S1.** Parameters of interest for the proposed intramitochondrial-intracellular functional genomic system.			
**Parameter function**	**Symbol**	**Value Range**	**Source**
Cellular ROS generation constant	*K1*	25–50 (nM day^{-1})	100%[SS]
Mitochondrial ROS generation	*K2*	120–240 (nM day^{-1})	(Gruber et al., 2011)[C]
ROS remediation constant	*K3*	0.1–0.5 (day^{-1})	(Giorgio et al., 2007)[SS]
skn-1 ROS-dependent activity coefficient	*K4*	60 (nM)	(Hoffman et al., 2017)[C]
daf-16 ROS-dependent activity coefficient	*K5*	75 (nM)	(Hoffman et al., 2017)[C]
sod-2 production constant	*K6*	1	(Gruber et al., 2011)[O]
UPR^{mt} activity coefficient (ROS_{mt}-based)	*K7*	40–60 nM	(Nelson et al., 2013)[C]
UPR^{ER} activity coefficient (BiP-based total)	*K8*	triangular(0.5,0.75,1)	100%[SS]
PERK phosphorylation coefficient	*K9*	0.015 (day^{-1})	(Halliday et al., 2017)[A]
skn-1-controlled mTOR activity coefficient	*K10*	2.0	(Robida-Stubbs et al., 2012)[A]
bec-1 production constant	*K11*	0.25 (% day^{-1})	100%[SS]
pink-1 activity constant	*K12*	1.0	(Zhang et al., 2017)[A]
PERK protein production constant	*PP*	1 (% day^{-1})	100%[SS]
Total PERK degradation constant	*PE*	0.1 (day^{-1})	100%[SS]
Mitochondrial ROS elimination constant	*kMRE*	0.2–2.0 (day^{-1})	(Gruber et al., 2011)[C]
Mitochondrial tau activity coefficient	*TM*	200 (%)	(Cenini and Voos, 2016)[O]
Cellular tau activity coefficient	*TC*	300–400 (%)	(Alavi Naini and Soussi-Yanicostas, 2015)[A]
Mutation fraction related to tau production	*mF*	0.5–1.5	(Fatouros et al., 2015)[A]
ROS-dependent tau conversion constant	*RT*	50 (nM)	(Dukan et al., 2000; Hoffman et al., 2017)[A,C]
mTOR-driven bec-1 inhibition constant	*MB*	1.5	(Antikainen et el., 2017)[A]
UPR^{ER}-mediated bec-1 increase constant	*UB*	1.5	(Koh et al., 2018)[A]
Max. ROS-based increase in UPR^{mt} activity	*KMT*	1.0	(Shpilka and Haynes, 2018)[A]

[SS] Optimized in the simulation to achieve the reported unstressed physiological steady-state percentage or range.
[O] Individually optimized value, generated using AnyLogic calibration to best emulate reported kinetic curves.
[A] Quantitated assumption based on qualitative evidence.
[C] Units converted to adhere to model standards.

Box S2: Equations pertaining to cell-level biochemical kinetics and activity nodes.

Differential equations governing (i) time-dependent cellular ROS accumulation and elimination (Gruber et al., 2011; Hoffman et al., 2017), (ii) PERK phosphorylation events (Halliday et al., 2017), (iii) tau protein production, aggregation and elimination, (iv) bec-1 protein content, and (v) UPR^{mt}-controlled hsp-60 content (Nargund et al., 2012):

$$\frac{d[ROS_{cell}]}{dt} = ROS_{mt} + K1\left(1-\left(\frac{skn1^3}{skn1^3+1.5^3}\right)\right)\left(\frac{Tau_{Agg}^5}{Tau_{Agg}^5+TC^5}\right) - K3*ROS_{cell}\left(\frac{sod2}{sod2+1.5}\right)$$

$$\frac{PERK}{dt} = PP - PERK*K9\left(\frac{Tau_{Agg}^3}{Tau_{Agg}^3+1/2\ *TC^3}\right)$$

$$\frac{pPERK}{dt} = PERK*K9\left(\frac{Tau_{Agg}^3}{Tau_{Agg}^3+1/2*TC^3}\right) - PE*pPERK$$

$$\frac{Tau}{dt} = 100\%*mF*\left(2-\frac{pPERK}{pPERK+1.5}\right) - \left(Tau\left[\frac{UPR}{UPR+1.5}\right]*\ hsp60*\frac{1}{\%_{hsp60}\ day}\right) - uniform(15{,}30)*Tau\left(\frac{ROS_{cell}^3}{ROS_{cell}^3+RT^3}\right)$$

$$\frac{Tau_{Agg}}{dt} = uniform(15{,}30)*Tau\left(\frac{ROS_{cell}^3}{ROS_{cell}^3+RT^3}\right) - \left(\frac{bec1}{bec1+50}\right)\ *\ Tau\_Agg$$

$$\frac{bec1}{dt} = 0.5\ +\ k11*\left(\frac{UPR^5}{UPR^5+UB^5}\right) - bec1\left(\frac{mTOR^{1.5}}{mTOR^{1.5}+MB}\right)$$

$$\frac{hsp60}{dt} = 1+\frac{UPRmt}{UPRmt+1}*triangular(1, 1.5, 2) - \frac{1}{day}*hsp60$$

Dynamics and normalized activity of reporter genes and responses involved in the molecular signature, dictated by Hill dynamics as opposed to Boolean functions. Hill dynamics theorized here and elsewhere have been mathematically optimized (as previously described in Hoffman et al., 2018) based on each node's corresponding literature information seen in Table S1. All corresponding assumptions are stated as well. KD and KS are fractional parameters that can be altered for genetic ablation experiments (basal = 1):

$$\frac{d[UPR^{mt}]}{dt} = 1\ +\ KMT*\left(\frac{ROS_{mt}^4}{ROS_{mt}^4+K7^4}\right) - \left(\frac{Tau_{Agg}^2}{Tau_{Agg}^2+TC^2}\right)$$

$$\frac{d[BiP]}{dt} = \frac{d[UPR]}{dt} = 1-K8\left(\frac{Tau_{Agg}^4}{Tau_{Agg}^4+2*TC^4}\right)$$

$$\frac{d[sod2]}{dt} = K6*UPRmt*daf16$$

$$\frac{d[daf16]}{dt} = KD\left[1+\left(\frac{ROS_{cell}^8}{ROS_{cell}^8+K5^8}\right)\right]$$

$$\frac{d[skn1]}{dt} = KS\left[1+\left(\frac{ROS_{cell}^2}{ROS_{cell}^2+K4^2}\right)\right]$$

$$\frac{d[pink1]}{dt} = 2-mTOR*K12$$

$$\frac{d[mTOR]}{dt} = 1.5-\left(\frac{skn1^2}{skn1^2+K10^2}\right)$$

Box S3: Agent-based methods applied for cellular vulnerability phenotyping.

As the ultimate outputs of the model, age-dependent cellular phenotype percentages are captured based on each cell's conditions of the dynamic response network. These conditionally-bound phenotypes are created using agent-based "state chart" functions, and are based on characteristically bifurcated conditions that arose from the bistability analyses performed. Transitions indicate the movement through several phenotypes, as depicted in Figure S2B. Cells first move through either possessing UPR^{ER} or UPR^{mt} deficiencies, and then both; once both deficiencies have been achieved from the aging process, neurons can then be rendered vulnerable to the effects of high ROS levels and tau protein aggregates. Once this final conditionally-bound vulnerability state is achieved, they then have a stochastic time requirement to activate stress responses and rescue from these deleterious conditions or they will be removed from the simulation to emulate cell death. These model developments are substantiated by the findings that UPR^{mt} activation promotes longevity and phenotypic resilience to ROS- and aggregation-mediated neurodegenerative outcomes (Cooper et al., 2017; Wu et al., 2018). These model developments are also substantiated by the fact that total UPR^{ER} activation is beneficial in preserving resilient cell phenotypes due to autophagic control and proteostatic measures, while the PERK translational branch upon aggregate-dependent overactivation leads to vulnerability (Halliday et al., 2017).

Transitions of increasing stress states within the agent-based framework (T1-T5), including the final optimized rate-dependent irreversible transition of full vulnerability to cell death (T6):

$$\boldsymbol{T1} : UPT^{mt}\_activity < 50\%$$

$$\boldsymbol{T2} : UPR\_activity < 50\%$$

$$\boldsymbol{T3} : UPR\_activity < 50\%, UPT^{mt}\_activity < 50\%$$

$$\boldsymbol{T4} : UPT^{mt}\_activity < 50\%, UPR\_activity < 50\%,$$

$$\boldsymbol{T5} : UPT^{mt}_{activity} < 50\%, UPR_{activity} < 50\%, pPERK > 150\%, ROS_{cell} > 100$$

$$\boldsymbol{T6} : exponential\ rate = cell\ death\ events\ per\ day = uniform(1, 2)$$

Transitions of reversible recovery from stress states within the agent-based framework (R1-R5):

$$\boldsymbol{R1} : UPT^{mt}_{activity} < 50\%, UPR_{activity} < 50\%, pPERK \leq 150\%, ROS_{cell} \leq 100$$

$$\boldsymbol{R2} : UPT^{mt}\_activity < 50\%, UPR\_activity \geq 50\%$$

$$\boldsymbol{R3} : UPT^{mt}\_activity \geq 50\%, UPR\_activity < 50\%$$

$$\boldsymbol{R4} : UPT^{mt}\_activity \geq 50\%, UPR\_activity \geq 50\%$$

$$\boldsymbol{R5} : UPT^{mt}\_activity \geq 50\%, UPR\_activity \geq 50\%$$

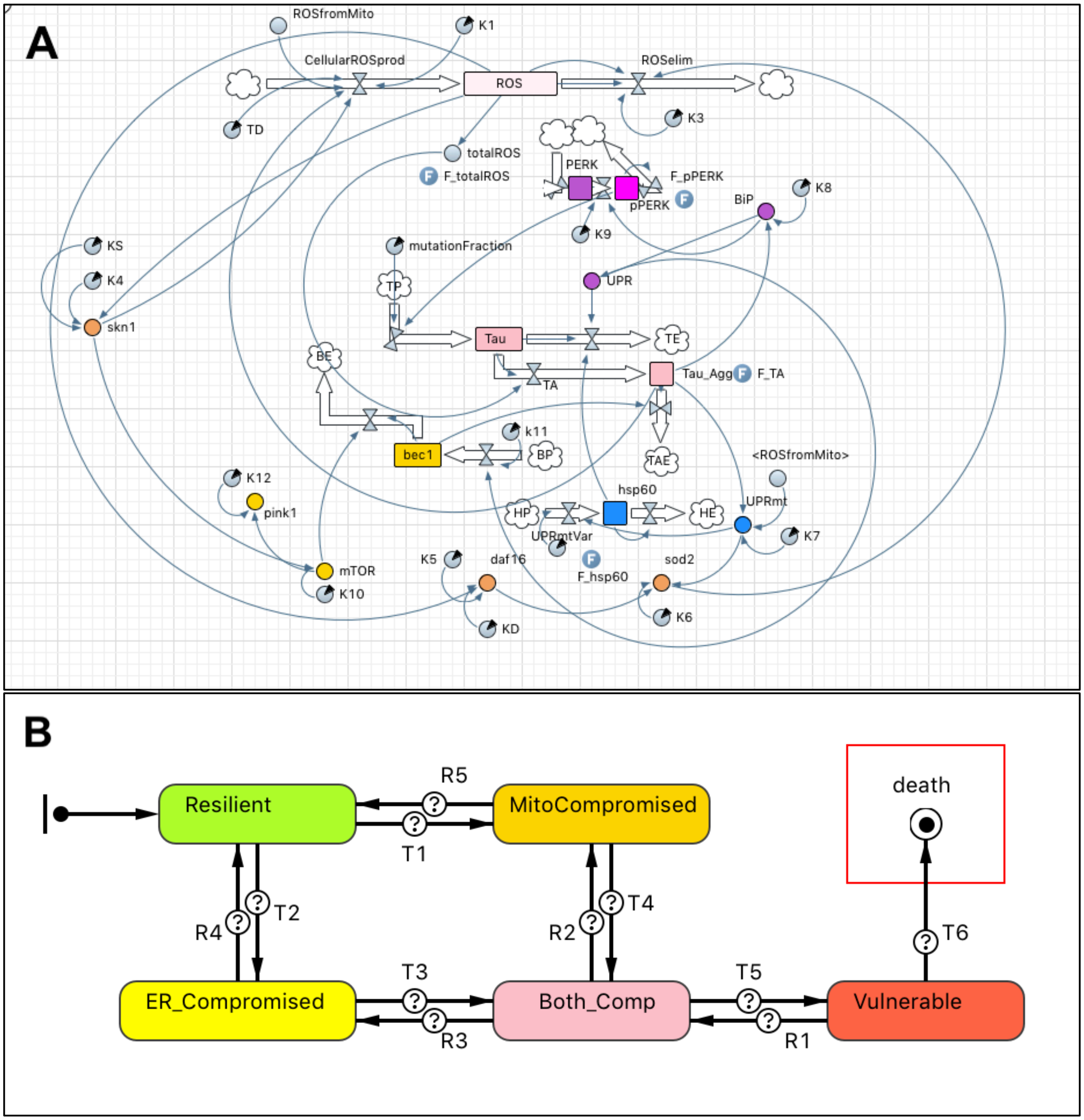

Figure S2. Simulation schematic displaying cell-level framework in the simulation software. **(A)** Deterministic equation-based network of defining cluster reporters and activators. Modeling components use the same symbolic structures as Figure S1, an all coefficients are described in the supplementary tables. Of importance to this figure, coefficients and variables with computational relationships to given stock compartments are denoted by long curved arrows. For example, *sod-2* expression, a dynamic variable in the schematic, is a positive regulator of ROS elimination. **(B)** Agent state chart with condition-based transitions leading to phenotypic agent states of vulnerability. Conditions for movement through states involve stress response activity loss, as defined in Box S3. Final state ("death") denotes simulated cell death, resulting in agent removal from the population.

4. Global sensitivity analysis

Parameters were tested for sensitivity within the network using a 5% value perturbation, as we have previously performed (Hoffman et al., 2017) and as it is described in the method section of the main article. The results of this analysis are shown in Table S2.

Table S2. Sensitivity analysis of cellular process parameters and stress response parameters within the molecular simulation network. Sensitivity coefficients (SC) with an absolute value greater than or equal to 1.0000 were deemed to have sensitive control over the system. SC values with an absolute value between 0.1000 and 0.9999 were considered for further analyses of system control. The response variable assessed for change in this analysis was cellular ROS content, as this drives many of the aging processes.

Parameter function	***Symbol***	***Early-life SC***	***Mid-life SC***	***Late-life SC***
Cellular ROS generation constant	*K1*	0.0422	0.2236	-0.1032
Mitochondrial ROS generation	*K2*	1.2348	0.4279	0.3418
ROS remediation constant	*K3*	-1.8216	-1.7145	-1.4484
skn-1 ROS-dependent activity coefficient	*K4*	0.0310	-0.1275	-0.2192
daf-16 ROS-dependent activity coefficient	*K5*	0.0000	0.0000	0.0000
sod-2 production constant	*K6*	0.0000	0.0000	0.0000
UPR^{mt} activity coefficient (ROS_{mt}-based)	*K7*	0.1112	-0.0740	-0.3841
UPR^{ER} activity coefficient (BiP-based total)	*K8*	0.0077	-0.0435	-0.4232
PERK phosphorylation coefficient	*K9*	0.0141	-0.0638	-0.6431
skn-1-controlled mTOR activity coefficient	*K10*	0.0021	-0.0638	-0.1759
bec-1 production constant	*K11*	0.0113	-0.3318	-0.2969
pink-1 activity constant	*K12*	0.0443	-0.0314	0.4873
PERK protein production constant	*PP*	0.0310	-0.1106	-0.6459
Total PERK degradation constant	*PE*	0.0471	-0.1151	-0.0226
Mitochondrial ROS elimination constant	*kMRE*	-0.6424	-0.4813	-0.5626
Mitochondrial tau activity coefficient	*TM*	-0.9069	-0.5100	-0.5084
Cellular tau activity coefficient	*TC*	-0.1949	-0.3889	-0.1930
ROS-dependent tau conversion constant	*RT*	-1.9560	-1.3090	-0.4021
mTOR-driven bec-1 inhibition constant	*MB*	-0.0134	-0.0595	-0.4529
UPR^{ER}-mediated bec-1 increase constant	*UB*	0.0303	-0.0595	-0.1095
Max. ROS-based increase in UPR^{mt} activity	*KMT*	0.0091	-0.3336	-0.0322

5. Inclusions of aberrant tau accumulation

To emulate tau-expressing anti-aggregating and pro-aggregating models within *C. elegans* (Fatouros et al., 2015) so that this simulation can be easily translatable in future nematode-based tau experiments, expressions governing tau production, accumulation and degradation have been made computationally malleable. For the simulation experiments of this study, the mutation fraction within the anti-aggregating cell populations (tau control) was set at 0.4, whereas the value for the pro-aggregating group was given a 100% increase at 1.4. The processes within the simulation controlled by tau content are primarily rooted in cellular and mitochondrial oxidative stress signaling and also notably involve the UPR^{ER} activities of the BiP and p-PERK branches (expressions included in Box S1 and Box S2).

References

Alavi Naini SM, Soussi-Yanicostas N (2015) Tau Hyperphosphorylation and Oxidative Stress, a Critical Vicious Circle in Neurodegenerative Tauopathies? *Oxidative medicine and cellular longevity*, *2015*, 151979. http://doi.org/10.1155/2015/151979

Antikainen H, Driscoll M, Haspel G, Dobrowolski R (2017) TOR-mediated regulation of metabolism in aging. *Aging cell*, *16*(6), 1219–1233. http://doi.org/10.1111/acel.12689

Cenini G, Voos W (2016) Role of Mitochondrial Protein Quality Control in Oxidative Stress-induced Neurodegenerative Diseases. Curr Alzheimer Res. 13(2): 164–173. https://www.ncbi.nlm.nih.gov/pubmed/26391041

Cooper JF, Machiela E, Dues DJ, Spielbauer KK, Senchuk MM, Van Raamsdonk JM (2017) Activation of the mitochondrial unfolded protein response promotes longevity and dopamine neuron survival in Parkinson's disease models. *Scientific reports*, *7*(1), 16441. http://doi.org/10.1038/s41598-017-16637-2

Dong X, Milholland B, Vijg J (2016) Evidence for a limit to human lifespan. Nature. 538(7624): 257–259. http://doi.org/10.1038/nature19793

Dukan S, Farewell A, Ballesteros M, Taddei F, Radman M, Nyström T (2000) Protein oxidation in response to increased transcriptional or translational errors. *Proc. Natl. Acad. Sci. U. S. A.* **97**, 5746–5749. https://doi.org/10.1073/pnas.100422497

Fatouros C, Pir GJ, Biernat J, Koushika SP, Mandelkow E, Mandelkow EM, Schmidt E, Baumeister R (2012) Inhibition of tau aggregation in a novel Caenorhabditis elegans model of tauopathy mitigates proteotoxicity. *Human Molecular Genetics.* **21**(16), 3587–3603. http://doi.org/10.1093/hmg/dds190

Fiorese CJ, Haynes CM (2017) Integrating the UPR^{mt} into the mitochondrial maintenance network. *Critical reviews in biochemistry and molecular biology*, *52*(3), 304–313. http://doi.org/10.1080/10409238.2017.1291577

Giorgio M, Trinei M, Migliaccio E, Pelicci PG (2007) Hydrogen peroxide: a metabolic by-product or a common mediator of ageing signals?. *Nature Rev. Mol Cell Biol.* **8**, 722–728. https://doi.org/10.1038/nrm2240

Halliday M, Radford H, Zents KAM, Molloy C, Moreno JA, Verity NC, Smith E, Ortori CA, Barnett DA, Bushell M, Mallucci GR (2017) Repurposed drugs targeting eIF2α-P-mediated translational repression prevent neurodegeneration in mice. *Brain.* **140**(6), 1768–1783. http://doi.org/10.1093/brain/awx074

Hoffman TE, Barnett KJ, Wallis L, Hanneman WH (2017) A multimethod computational simulation approach for investigating mitochondrial dynamics and dysfunction in degenerative aging. *Aging Cell.* **16**, 1244–1255. http://doi.org/10.1111/acel.12644

Hoffman TE, Acerbo ER, Carranza KF, Gilberto VS, Wallis LE, Hanneman WH (2018) Ultrasensitivity dynamics of diverse aryl hydrocarbon receptor modulators in a hepatoma cell line. Archives of Toxicology. [Epub December 2018] https://doi.org/10.1007/s00204-018-2380-z

Kaufman DM, Wu X, Scott BA, Itani OA, Van Gilst MR, Bruce JE, Crowder CM (2017) Ageing and hypoxia cause protein aggregation in mitochondria. *Cell death and differentiation*, *24*(10), 1730–1738. http://doi.org/10.1038/cdd.2017.101

Koh JH, Wang L, Beaudoin-Chabot C, Thibault G (2018) Lipid bilayer stress-activated IRE-1 modulates autophagy during endoplasmic reticulum stress. *J Cell Sci.* 131(22). pii: jcs217992. http://doi.org/10.1242/jcs.217992

Nargund AM, Pellegrino MW, Fiorese CJ, Baker BM, Haynes CM (2012) Mitochondrial import efficiency of ATFS-1 regulates mitochondrial UPR activation. *Science.* **337**, 587–590. https://doi.org/10.1126/science.1223560

Nelson CJ, Li L, Jacoby RP, Millar AH (2013) Degradation rate of mitochondrial proteins in Arabidopsis thaliana cells. *J. Proteome Res.* **12**, 3449–3459. https://doi.org/10.1021/pr400304r

Pharaoh G, Pulliam D, Hill S, Sataranatarajan K, Van Remmen H (2016) Ablation of the mitochondrial complex IV assembly protein Surf1 leads to increased expression of the UPR(MT) and increased resistance to oxidative stress in primary cultures of fibroblasts. *Redox biology*, *8*, 430–438. http://doi.org/10.1016/j.redox.2016.05.001

Robida-Stubbs S, Glover-Cutter K, Lamming DW, Mizunuma M, Narasimhan SD, Neumann-Haefelin E, Sabatini DM, Blackwell TK (2012) TOR signaling and rapamycin influence

longevity by regulating SKN-1/Nrf and DAF-16/FoxO. *Cell Metab.* **15**, 713–724. https://doi.org/10.1016/j.cmet.2012.04.007

Schwab JD, Siegle L, Kühlwein SD, Kühl M, Kestler HA (2017) Stability of Signaling Pathways during Aging-A Boolean Network Approach. *Biology*, *6*(4), 46. http://doi.org/10.3390/biology6040046

Shpilka T, Haynes CM (2018) The mitochondrial UPR: mechanisms, physiological functions and implications in ageing. Nat Rev Mol Cell Biol. 19(2): 109–120. http://doi.org/10.1038/nrm.2017.110

Stroustrup N, Anthony WE, Nash ZM, Gowda V, Gomez A, López-Moyado IF, […] Fontana W (2016) The temporal scaling of Caenorhabditis elegans ageing. *Nature*. *530*(7588), 103–107. http://doi.org/10.1038/nature16550

Verlingue L, Dugourd A, Stoll G, Barillot E, Calzone L, Londoño-Vallejo A (2016) A comprehensive approach to the molecular determinants of lifespan using a Boolean model of geroconversion. *Aging Cell.* **15**, 1018–1026. https://doi.org/10.1111/acel.12504

Wu Z, Senchuk MM, Dues DJ, Johnson BK, Cooper JF, Lew L, […] Van Raamsdonk JM (2018) Mitochondrial unfolded protein response transcription factor ATFS-1 promotes longevity in a long-lived mitochondrial mutant through activation of stress response pathways. *BMC biology*, *16*(1), 147. http://doi.org/10.1186/s12915-018-0615-3

Zhang Y, Nguyen DT, Olzomer EM, Poon GP, Cole NJ, Puvanendran A, Phillips BR, Hesselson D (2017) Rescue of Pink1 Deficiency by Stress-Dependent Activation of Autophagy. *Cell Chem Biol.* 24(4):471–480.e1–e4 http://doi.org/10.1016/j.chembiol.2017.03.005
